# Exposure of Midge Larvae (*Chironomus riparius*) to Graphene Oxide Leads to Development Alterations

**DOI:** 10.3390/toxics10100588

**Published:** 2022-10-05

**Authors:** Lauris Evariste, Laura Lagier, Chloé Chary, Antoine Mottier, Stéphanie Cadarsi, Eric Pinelli, Emmanuel Flahaut, Laury Gauthier, Florence Mouchet

**Affiliations:** 1 Laboratoire d’Ecologie Fonctionnelle et Environnement, UMR 5245 CNRS, Université Paul Sabatier, 31062 Toulouse, France; 2 CIRIMAT, CNRS-INP-UPS, UMR N°5085, Université Toulouse 3 Paul Sabatier, 118 Route de Narbonne, 31062 Toulouse, France

**Keywords:** chironomids, aquatic ecotoxicology, nano-ecotoxicology, graphene, biomarkers

## Abstract

Despite the fast-growing use and production of graphene-based nanomaterials (GBMs), data concerning their effects on freshwater benthic macroinvertebrates are scarce. This study aims to investigate the effects of graphene oxide (GO) on the midge *Chironomus riparius*. Mortality, growth inhibition, development delay and teratogenicity, assessed using mentum deformity analysis, were investigated after a 7-day static exposure of the first instar larvae under controlled conditions. The collected data indicated that the survival rate was not impacted by GO, whereas chronic toxicity following a dose-dependent response occurred. Larval growth was affected, leading to a significant reduction in larval length (from 4.4 to 10.1%) in individuals reaching the fourth instar at any of the tested concentrations (from 0.1 to 100 mg/L). However, exposure to GO is not associated with an increased occurrence of mouthpart deformities or seriousness in larvae. These results highlight the suitability of monitoring the larval development of *C. riparius* as a sensitive marker of GO toxicity. The potential ecological consequences of larval size decrease need to be considered for a complete characterization of the GO-related environmental risk.

## 1. Introduction

Graphene-family materials (GFMs), including 2D nanomaterials such as pristine graphene or its oxidized form graphene oxide (GO), exhibit unique properties triggering interest in many areas [1]. Indeed, the use of GO for industrial processes is promoted by its stable dispersion capacity in water or organic solvents [2,3], facilitating its incorporation in various matrices [4]. In addition, due to its high adsorption capacity, GO-based applications are developed for water pollution remediation [5,6,7,8]. Concomitantly with the development of new technological and industrial applications, the improvement of production processes leads to an increase in the annual production of GO, which is expected to reach the industrial scale within a few years [9,10]. For these reasons, GO is likely to be released into the environment at each step of its life cycle (production, use and recycling) [11,12,13], especially in the aquatic environment which is known to act as a sink for nanoparticles [14]. Although no current information regarding the environmental concentration of GBMs is available due to technological limitations [15], it was estimated that GO would reach an environmental concentration similar to that of carbon nanotubes, which was estimated to range from 0.001 to 1000 µg/L based on dynamic probabilistic modeling [16,17]. The presence of these nanomaterials in the aquatic environment constitutes a potential hazard for the organisms living in these ecosystems.

Compared to the increasing number of studies dealing with the synthesis and applications of GO, relatively few studies devoted to the study of its toxicity, especially in the aquatic environment [18,19]. While GO toxicity towards organisms from lower trophic levels such as bacteria or algae is more documented [20,21,22,23], studies dealing with GO toxicity towards aquatic invertebrates are less numerous and indicated GO accumulation and the induction of oxidative stress in model organisms such as *Daphnia magna* [24,25]. However, the available data on invertebrates are not fully representative of the different ecological niches covered by invertebrate species. Considering benthic organisms such as chironomids, GO was found to exert weak toxicity, as indicated by the absence of mortality following exposure to concentrations of up to 100 mg/L [26]. A recent study indicated that an exposure of *Chironomus riparius* larvae to GO for up to 96 h induced oxidative stress and lipid peroxidation, as indicated by the induction of antioxidant enzymatic activities and malondialdehyde levels [27]. However, no study was devoted to determining the effects on larval development and phenotypic consequences of a GO exposure. Thus, a more complete evaluation of the ecotoxic potential of GBMs, including GO, is needed to ensure a safer use of these nanomaterials.

In this context of limited knowledge about the ecotoxicity of GO, the macroinvertebrate *Chironomus* sp. constitutes a relevant model species for GO ecotoxicological assessment. Indeed, chironomids are sentinel species that are widely used as indicators of freshwater ecosystem quality [28,29] or for the ecotoxicological assessment of polluted sediment or water [30,31,32]. In addition, they were shown to be sensitive to nanoparticles, the exposure to which led to both physiological and behavioral impairments [33,34,35,36,37]. From an ecological point of view, chironomid larvae are benthic organisms that play a key role in the environment through direct or indirect effects on organic matter recycling, nitrogen cycling or bioturbation [38,39,40], while constituting an important food source for organisms of higher trophic levels such as fishes [41] and birds [42]. Thus, physiological impairments or alteration of the chironomid population structure can have deleterious consequences on ecosystem functioning.

Thus, the aim of this work is to determine the consequences of GO exposure towards *C. riparius*, focusing on development alterations, in order to better characterize the hazards of GO towards this species and to help better predict the potential consequences for other organisms or ecosystem functioning.

## 2. Materials and Methods

### 2.1. Synthesis and Characterization of Graphene Oxide (GO)

Graphene oxide was provided by Antolin Group and prepared by oxidation of Grupo Antolin Carbon Nanofibers (GANF) using Hummer’s method [43,44]. GO characteristics were previously described by Lagier and collaborators [45]. Briefly, data of elemental analysis were obtained using X-ray photoelectron spectroscopy analysis to determine the atomic percentage of C and O (69.7 and 30.3 at. %, respectively). The specific surface area was 206 m^2^/g, according to Brunauer, Emett and Teller’s theory (BET), on the powdered sample of GO. The number of layers was estimated to be from 1 to 5 from high-resolution transmission electron microscopy (HRTEM). Lateral dimensions from 0.2 to 8 µm were also assessed by TEM.

### 2.2. GO Physical Dispersion before Contamination

The desired quantity of GO powder was first weighed and dispersed for 30 min in an ultrasonic bath (Bioblock 89863, typ 570 HF Freq 35 kHz) in deionized water to obtain a stock suspension (5 mg/mL^−1^). Then, depending on the desired concentration, required amounts of this suspension were introduced in individual glass test tubes and adjusted to 20 mL by the addition of deionized water. For each concentration, 7 test tubes of 20 mL were thus prepared, corresponding to the number of exposure replicates.

### 2.3. Chironomus Rearing

*Chironomus* breeding was maintained in the laboratory according to the norms [46,47] in a thermostated room at 21 ± 1 °C, following a 16–8 h light–dark cycle in 20 L aquaria. The culture water was reconstituted (RW) with deionized water and salts (66.2 mg/L CaCl_2_, 2H_2_O; 61.4 mg/L MgSO_4_, 7H_2_O; 96 mg/L NaHCO_3_; 4 mg/L KCl; 63 mg/L CaSO_4_, 2H_2_O; 1 mg/L NaBr), aerated and maintained at a depth of around 20 cm. Sand of fine grain size (<350 µm) from Fontainebleau (VWR, Fontenay-sous-Bois, France) was used as substrate (3 cm). *Chironomus* were fed daily with 400 mg per tank of finely crushed fish food (Tetramin^®^), dispersed in distilled water (15 min at ultrasonic bath; Bioblock 89863, typ 570 HF Freq 35 kHz).

A few days before the exposure, egg masses collected from the aquaria on the same day of hatching were placed in crystallizing dish containing 1 L of RW, a fine layer of sand, and 150 mg of Tetramin^®^. Young 48 h old larvae (first instar) were then collected for the exposure experiment.

### 2.4. Experimental Design and Exposure Conditions

Following the normalized procedure [46,47], *Chironomus* larvae were exposed to increasing GO concentrations (0, 0.1, 1, 10 and 100 mg/L) under static conditions (total volume of 300 mL) for 7 days to allow larvae to reach the last larval instar. Prior to exposure, 7 glass beakers (Pyrex) per condition were prepared, corresponding to 7 replicates. Each beaker was filled with 75 mL of sand, 265 mL of RW and 35 mL of GO dispersion in RW (only RW for the control group), maintained under gentle aeration. On the first day of exposure, ten 48 h old *Chironomus* larvae (mean length of 1.54 ± 0.04 mm) were introduced into each beaker by pipetting, leading to a total of 70 larvae used per experimental condition. Aeration was interrupted during *Chironomus* introduction in order to allow them to sink more easily. Then, contamination was performed, consisting in the introduction of extemporaneously dispersed GO (2 min in an ultrasonic bath). The negative control (Ctrl) containing sediment and RW alone was also achieved in 7 replicates. Each beaker received 1 mg of finely ground Tetramin^®^ daily. Aeration was restarted on the day following *Chironomus* introduction and continued until the end of the experiment. Water quality parameters, such as pH (7–8), temperature (21 ± 1 °C), nitrite, ammonia and dissolved oxygen content (9.4 ± 0.3 mg/L) were monitored throughout the experiment and did not vary between the tested conditions. Maximum nitrite and ammonia concentrations measured were 0.23 mg/L and 4.62 mg/L, respectively, which are lower than the maximum thresholds for the normalized test (5 mg/L for nitrites and 10 mg/L for ammonia) [47] and are not harmful to larvae and their development [48].

### 2.5. Data Acquisition

#### 2.5.1. Survival Rate

At the end of exposure, the number of living chironomids in each experimental unit was determined to calculate the survival rates compared to the number of chironomids initially introduced. The test is considered valid if the survival rate calculated for the negative control condition is higher than 70% [47].

#### 2.5.2. Growth Measurement

At the end of exposure, surviving larvae were stored in ethanol (70%) until data processing. Larvae photographs were collected under a binocular magnifier (Olympus SZX7) equipped with a photo camera (Olympus E-620) prior to length measurement using ImageJ software calibrated with a millimetric scale. In order to determine the potential growth inhibition, normalized growth rate (NGR) was calculated based on previous work [49] as follows:

$$NGR\;\left(\%\right)=\left(\frac{Ld7-MLd0}{MLd0}\times 100\right)\times \left(\frac{100}{MLCtrld7}\right)$$

where *Ld*7 corresponds to the length of one larva at the end of the exposure, *MLd*0 is the mean length at day 0 of larvae from the exposure condition and *MLCtrld*7 is the mean length of larvae from the negative control at the end of the exposure.

#### 2.5.3. Determination of Development Delay

The development stage reached by each of the collected surviving *Chironomus* larvae was determined under a binocular magnifier based on the width measurement of cephalic capsules using ImageJ software. Thus, larvae with cephalic capsule width ranging from 0.07 to 0.12 mm are classified as stage 1; from 0.13 to 0.24 mm, as stage 2; from 0.26 to 0.40 mm, as stage 3; and from 0.43 to 0.60 mm, as stage 4 [46]. In each treatment group, the proportion of larvae reaching stage 4 was compared to the Ctrl group.

#### 2.5.4. Teratogenicity Assessment

Teratogenicity induced by GO was evaluated through the analysis of chironomid mentum deformities following the 7 days of exposure. Such deformities can appear after every larval molt occurring between each development instar in the presence of a teratogenic compound. Thus, only cephalic capsules from 4th instar larvae were considered for teratogenicity assessment to integrate the potential effects occurring after three successive molts.

Sample preparation was performed as follows: cephalic capsules were first discolored with potassium hydroxide (15%) at 95 °C for 12 min prior to potassium hydroxide being replaced by ethanol (70%) for 12 h to stop the reaction. Then, capsules were mounted on a microscope slide and fixed with Quick-hardening mounting medium (Sigma Aldrich, Saint-Quentin-Fallavier, France).

**Figure 1:**
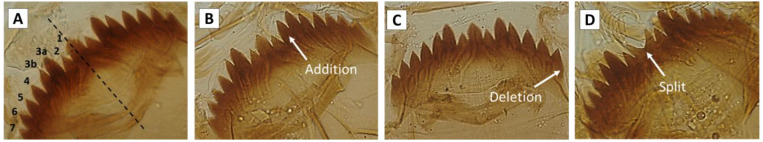
Microscopic observations of the mentum of Chironomus larvae (× 400). (**A**) Non-deformed mentum; teeth are numbered from 1 to 7 following a symmetry axis indicated by the dashed black line. (**B**) Tooth addition (between No. 3b and 4). (**C**) Tooth deletion (No. 7). (**D**) Split of central tooth.

Cephalic capsules were observed under a light microscope, and mentum anomalies were classified according to the work of Warwick and Tisdale [50]. Blind analysis was performed, and as previously suggested, only anomalies classified as tooth deletions, additions and Khön gaps (not observed during the experiment) or first tooth split were considered to avoid analytical inconsistencies [51] (Figure 1A–D). To evaluate the severity of the anomalies in affected larvae, a scoring was used based on the previous work from Vermeulen and collaborators [52].

### 2.6. Integrated Biomarker Response (IBR)

The integrated biomarker response index (IBR) [53], modified according to Devin and collaborators [54], was calculated using the biological endpoints measured in *C. riparius*. The IBR allows the integration of all the measured responses to an integrative index in order to describe biomarker variations; i.e., stress levels, in the different experimental conditions and simplify the interpretation of the data.

IBR is calculated using Microsoft Excel software according to the following steps:

(1) Data are standardized through the calculation of Y = (X − *m*)/*s*, where X is the mean value for the biomarker at a given treatment and *m* and *s* are the general mean and the standard deviation of all data from the biomarker considered, respectively.

(2) A score S = Z + |min|, where S ≥ 0; Z is computed as Y x −1 or 1 depending on the inhibition or activation, respectively, of the considered biomarker; and |min| = absolute minimum value of Y for the considered biomarker.

(3) The Si values are then plotted on a radar graph.

(4) The IBR values for each experimental condition are calculated as follows:

$$\mathrm{IBR}=\sum_{i=1}^{n}Ai$$

where Ai=Si × Si+1×sinα.

*S_i_* and *S_i+1_* represent two consecutive clockwise biomarker scores from the radar graph, *A_i_* is the area that connects two scores, *k* is the number of biomarkers considered for the analysis and α = 2π/*k*.

### 2.7. Statistical Analysis

Graphs were generated and statistical analysis was performed using the software GraphPad Prism 9.3.1. Data are presented as mean ± standard error of the mean (SEM), considering each glass beaker as an experimental unit (*n* = 7 per condition). Data related to *C. riparius* biological responses such as survival rate, growth rate, development delay, larval length and occurrence of mentum deformities were analyzed using one-way analysis of variance (ANOVA) followed by Tukey test after verifying that the assumptions of normality and homogeneity of variance were met. Statistical analysis of the severity score of mentum deformities was performed using Kruskal–Wallis as the number of replicates for this endpoint was lower due for example to an absence of larvae reaching stage 4 in some experimental replicates following exposure to high GO concentrations or to an absence of deformities in replicates from some experimental replicates.

## 3. Results and Discussion

### 3.1. GO Exposure and Survival Rate

**Figure 2:**
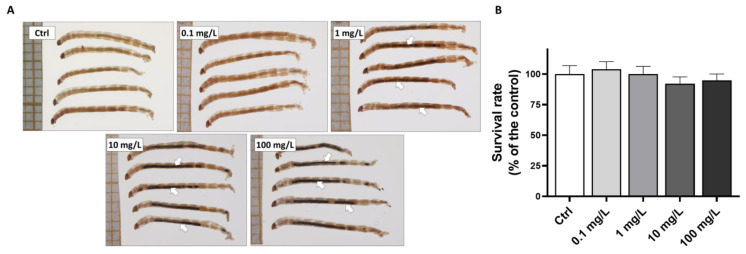
Macroscopic observations of *C. riparius* whole bodies following 7 days of exposure to GO at concentrations of 0, 0.1, 1, 10 and 100 mg/L (**A**) and associated survival rate (**B**). Arrows indicate the presence of graphene oxide in the digestive tract of exposed chironomids.

At the end of the 7 days of exposure, accumulation of GO is observed within the digestive tract of chironomids exposed to concentrations from 1 to 100 mg/L (Figure 2A), confirming the bioavailability of the nanomaterials at the sediment–water interface following contamination via the water column. The survival rate calculated in the control group (Ctrl) reached 84.6 ± 5.4% (59 individuals collected for this condition over the 70 initially introduced) which is over the 70% recommended [47], validating the use of the data from this experiment. No significant decrease in chironomid survival was noticed in the presence of GO up to 100 mg/L (ANOVA, *p* = 0.669) (Figure 2B).

The bioavailability of GO for the organisms living in the sediment is mostly determined by its fate in aquatic ecosystems. While GO exhibits a great dispersion stability in deionized water [55], GO aggregation and sedimentation were previously observed in closely related salt-containing exposure media [36,45]. This phenomenon was suggested to be associated with interactions between ions from the media, including Ca^2+^ or Na^+^, and the functional groups present at the GO surface [56,57]. Indeed, positive ions could adsorb on the negatively charged functional groups, leading to sedimentation of the nanomaterials. Thus, using transport modeling, it was suggested that the release of GO into an aquatic ecosystem would lead to long-term accumulation of the material in the sediment [58], increasing its bioavailability, i.e., hazard, for organisms living at the sediment interface such as chironomid larvae. In addition, due to *C. riparius* burrowing activities, settled GO was recovered within the sediment superficial layer, increasing its mobility within the substrate.

GBM accumulation in the digestive tract of multiple aquatic organisms living in different ecological niches, including vertebrates [45,59,60], pelagic invertebrates [24,61,62] and benthic invertebrates such as *C. riparius* [27], was previously observed. GO accumulation within the digestive tract of this species could favor the trophic transfer of GO towards secondary consumers feeding on this species.

In line with a previous study [27], our results indicate that the survival of *C. riparius* larvae is not affected by the GO exposure, even at a high dose; in contrast, the survival rate of other invertebrates such as *Artemia salina* and *Daphnia magna* was previously reported to be reduced at GO concentrations of 100 and 50 mg/L respectively [24,63]. These differences might be due to intrinsic physiological sensitivity differences between species but also due to the presence of sediment which protects against GO toxicity [64]. Compared to *Daphnia magna* which use gill respiration [65], oxygen is mainly provided by integumentary diffusion in *Chironomus* larvae, binding with hemoglobin, allowing larvae to survive in a low-oxygen environment. Thus, the possible gill clogging encountered in *D. magna* is less likely to impact the development of *C. riparius* larvae given the differences between the two species in respiratory physiology. Interestingly, the absence of significant mortality in the tested organisms exposed to GO will allow the detection of sublethal effects associated with nanomaterial exposure.

### 3.2. Effects of GO Exposure on Larval Growth and Development

**Figure 3:**
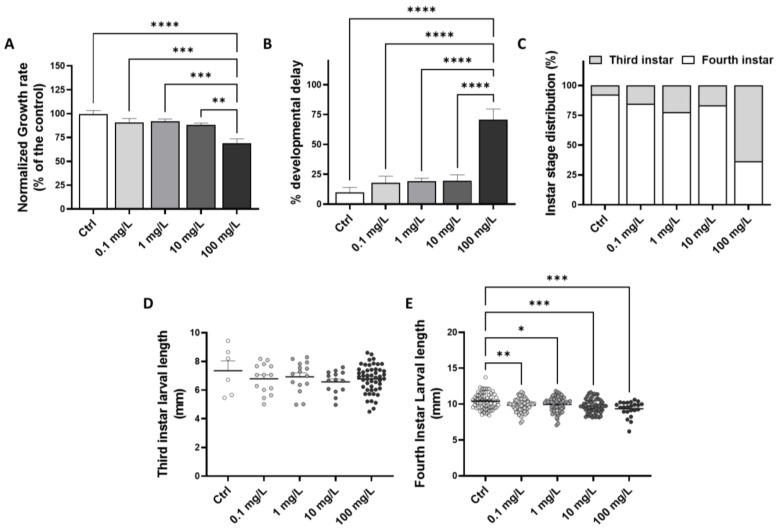
Effects of GO exposure on *C. riparius* growth rate (**A**), the occurrence of development delay (**B**) and development stage reached at the end of 7 days of exposure (**C**). Length of the larvae from third (**D**) and fourth (**E**) instar measured at the end of the exposure. Each dot represents an individual from the experimental condition. Statistical analysis was conducted using ANOVA, followed by post hoc Tukey test when *p* < 0.05. “*” indicates significant differences compared to the negative control (Ctrl) (*: *p* ≤ 0.05; **: *p* ≤ 0.01; ***: *p* ≤ 0.001; ****: *p* ≤ 0.0001).

Based on larval length measurement following the 7 days of exposure to GO, a significantly lower growth rate compared to the control group (−30.9%) was measured in the chironomid larvae exposed to the highest tested GO concentration of 100 mg/L (Figure 3A). Based on the developmental stage determined using cephalic capsule width measurement at the end of the exposure, it appears that compared to the control group where 90.2 ± 4.2% of the larvae reached the last development stage, the growth inhibition measured following exposure to GO at 100 mg/L is associated with a significant developmental delay with only 29.5 ± 9% reaching the fourth instar (ANOVA, *p* < 0.001) (Figure 3B). The collected data allowed the calculation of an EC_50_ of 38.74 mg/L, leading to the occurrence of developmental delay in 50% of the exposed larvae. While development is delayed by exposure to high GO doses, the larvae with altered development all reached stage 3 (Figure 3C), suggesting that the larval development is not fully compromised.

As larvae from the third instar are smaller compared to those from the fourth instar, the growth inhibition calculated is mainly due to the higher prevalence of larvae from stage 3 in the samples. However, when independently comparing the larval length from each stage to the control group, it appears that the larvae from stage 3 are of similar length between conditions (ANOVA, *p* = 0.591) (Figure 3D), while larvae from the fourth instar exhibit a significantly lower length compared to the control group in any experimental condition (ANOVA, *p* < 0.001) (Figure 3E). Thus, larvae reaching the last development stage in the presence of GO are significantly smaller compared to larvae from the control group.

Despite a slight growth rate decrease noticed for the overall larvae following exposure to GO concentrations from 0.1 to 10 mg/L, significant effects are only detected after exposure to GO at 100 mg/L. Other studies reported that chironomid larval growth may be affected by carbon-based nanoparticles such as fullerene or carbon nanotubes [66,67]. Similarly, growth inhibition was evidenced in other species such as the protozoan *Euglena gracilis* after 10 days of exposure to GO at concentrations ranging from 0.5 to 5 mg/L [68], as well as in vertebrate species, including amphibians [45].

In the present study, macroscopic observations demonstrate that the unselective feeding behavior of larvae causes them to ingest GO [69], as evidenced in their digestive tract. Thus, growth inhibition is associated with GO accumulation within the intestine, which may cause several toxicological mechanisms contributing to the observed effects. GO is known to exert antimicrobial activities [70], which are able to impair the gut microbiome of organisms [71]. In chironomids, the gut microbiota is known to protect the host from contaminants, while its impairment was associated with larval growth impairment in organisms exposed to contaminants [72,73,74]. In addition, as previously described in the case of carbon nanotubes [75], the presence of GO could sequestrate essential micronutrients such as amino acids, vitamins, nucleic acids and other macromolecules essential for larval growth. The hypothesis of growth impairment due to disturbances of the digestive physiology is supported by a study suggesting that food restriction in *C. riparius* is associated with a slower growth but remains to be evaluated in this particular case of GO exposure [76]. In addition, the toxic action of GO may be mediated by cellular membrane injuries causing oxidative stress that was previously shown to occur in chironomid larvae exposed to GO for 4 days at lower concentrations [27].

Along with growth inhibition, development delay is observed in larvae, leading to a decreased proportion of the fourth instar compared to larvae from the third instar. Thus, either the alteration of the energy harvest or the induction of oxidative stress may impair energy balance, leading to an increased energy allocation for detoxication while a decreased proportion is allocated for growth and development [77,78]. Development delay could in turn delay the emergence of adult *Chironomus*, which may have significant consequences at the population level. For instance, a decreased adult emergence may impact reproduction success, leading to a reduction in the genetic fitness of the population [79]. However, the deleterious effects on growth and development are not significant at the environmentally relevant lowest GO concentrations tested. Thus, the deleterious effects observed at high concentrations are unlikely to occur in natural environments.

Although this point needs to be evaluated, a significant decrease in larval size observed in presence of GO could potentially lead to adverse ecological consequences. Indeed, it was previously observed that the metamorphosis of smaller larvae gives rise to smaller adults, which may impact the survival rate [80]. In addition, it appears that smaller adult females of *C. riparius* exhibit a lower fecundity [81]. Thus, the GO-related size reduction of larvae observed following exposure to environmentally relevant concentrations could impair the population dynamics. In addition, the chironomid larval size constitutes a parameter that was shown to influence prey–predator relationships [42,82]. Thus, we can hypothesize that significant changes in the size distribution of chironomids could influence the rest of the food chain, modifying trophic interactions between species.

### 3.3. Effects of GO Exposure on the Induction of Mentum Deformities

**Figure 4:**
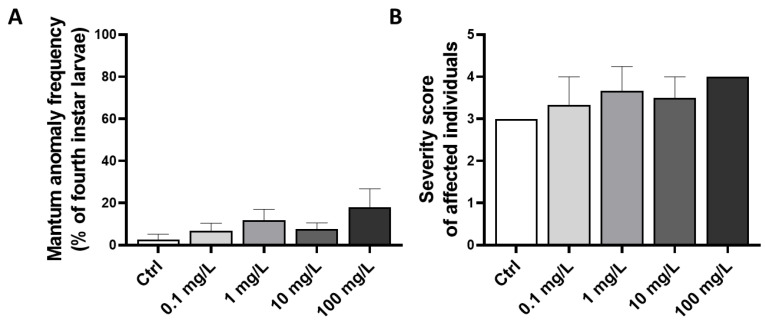
Effects of exposure to an increasing GO concentration on the frequency of mentum deformities occurrence in fourth instar *C. riparius* larvae (**A**) and severity score of affected larvae from each experimental condition (**B**). Statistical analysis was conducted using ANOVA for anomaly frequency and Kruskal–Wallis for severity score, *p* > 0.05.

The study of the presence of mentum deformities in *C. riparius* larvae indicated only a dose-dependent trend in the increase in anomaly frequency occurrence (ANOVA, *p* = 0.268), reaching up 18.1 ± 8.7 % in the stage 4 chironomids following exposure to the highest dose of GO (100 mg/L) (Figure 4A). Among larvae harboring an abnormal mentum, the severity of the deformities was not significantly increased by the increasing GO concentration (K-W, *p* = 0.769) (Figure 4B).

Teratogenicity refers to the deformities which may be found in different structures of the cephalic capsule of chironomids [50]. Such deformities are induced through the interference of a phenomenon during the molting [52], and they have to be distinguished from the normal wear or breakage of mouthparts, which occur due to feeding activities in coarse sediments. *Antennae* and several mouthparts, including, pecten, mandibles and mentum, may be affected by deformities, and each of them may provide a specific response profile to a contaminant [83,84]. Only the mentum was investigated in the present paper as it constitutes a widely used indicator of toxic stress providing a high sensitivity to contaminants [85]. Positive correlations between chironomid deformities and the degree of contamination have been established, but these relationships were only considered as qualitative [28]. On the basis of paleo-limnologic records, natural mentum deformity levels have been estimated to vary between 0 and 0.8% [86]. However, possibly due to the ubiquitous dispersion of contaminants in the environment, levels in sites considered as control sites currently vary between 0 and 8% [87], which is consistent with our results in the control group from our experiment.

In chironomid larvae, other studies report the induction of mouthpart deformities by various contaminants, including organic compounds [88] and metals [85,89,90], while the assessment of teratogenicity of carbon-based nanomaterials in this organism is limited yet. Since mentum deformities occur during molting which is regulated by hormones [88], our study suggests that GO exposure is unlikely to act as an endocrine disruptor towards *C. riparius* under our experimental conditions.

### 3.4. Integration of Biological Responses into the IBR Index

**Figure 5:**
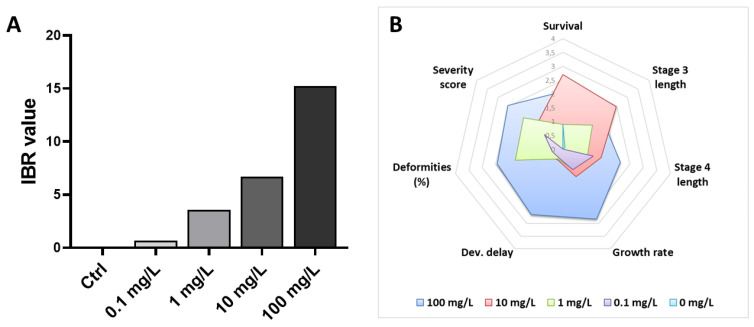
Integrated biomarker response index calculated based on *C. riparius* endpoints measured following 7 days of exposure to increasing GO concentrations (**A**). Representation of endpoint contributions to the calculated index for each experimental condition (**B**).

The data of seven endpoints monitored (survival, growth rate, development delay, third instar length, fourth instar length, teratogenesis frequency and severity) under the different exposure conditions were used to calculate IBR values. The obtained results indicated an increase in the calculated values in a monotonic dose-dependent manner, reaching the highest value after 7 days of exposure to GO at 100 mg/L (Figure 5A). The chart radars showed that changes in the IBR value were mostly associated with the growth rate and that fourth instar larval length and the occurrence of mentum deformities contributed to the overall calculated IBR score (Figure 5B). As previously suggested in other organisms, including worms, mollusks and fishes [91,92,93], the use of the IBR index seems to be an efficient tool when used with *C. riparius* for the evaluation of GBM toxicity, providing simplified results which are integrative of the overall biological responses to help regulatory agencies in evaluating the ecotoxic potential of these nanomaterials.

## 4. Conclusions

Chironomus larvae exposed to GO for seven days show no mortality or teratogenicity but a clear dose-dependent chronic toxicity leading to a markedly decreased length of larvae reaching the last larval instar, even when exposed to low doses. Integration into the IBR index of the information collected from the measured endpoints confirms that increasing GO exposure concentration leads to higher stress levels, attesting the relevance of chironomids as a model species to evaluate the ecotoxic impact of GO. Further investigations are still needed to underline the mechanistic effects associated with these development alterations through the use of other biomarkers, as well as to determine the ecological consequences of the observed effects as chironomid larvae are a key component of food webs in many aquatic ecosystems.
